# Supplementary figures and images for: Association between surgeon training grade and the risk of revision following unicompartmental knee replacement: An analysis of National Joint Registry data
Source: PLoS Med. 2024 Sep 10;21(9):e1004445. doi: 10.1371/journal.pmed.1004445 (PMC11386457; doi:10.1371/journal.pmed.1004445)

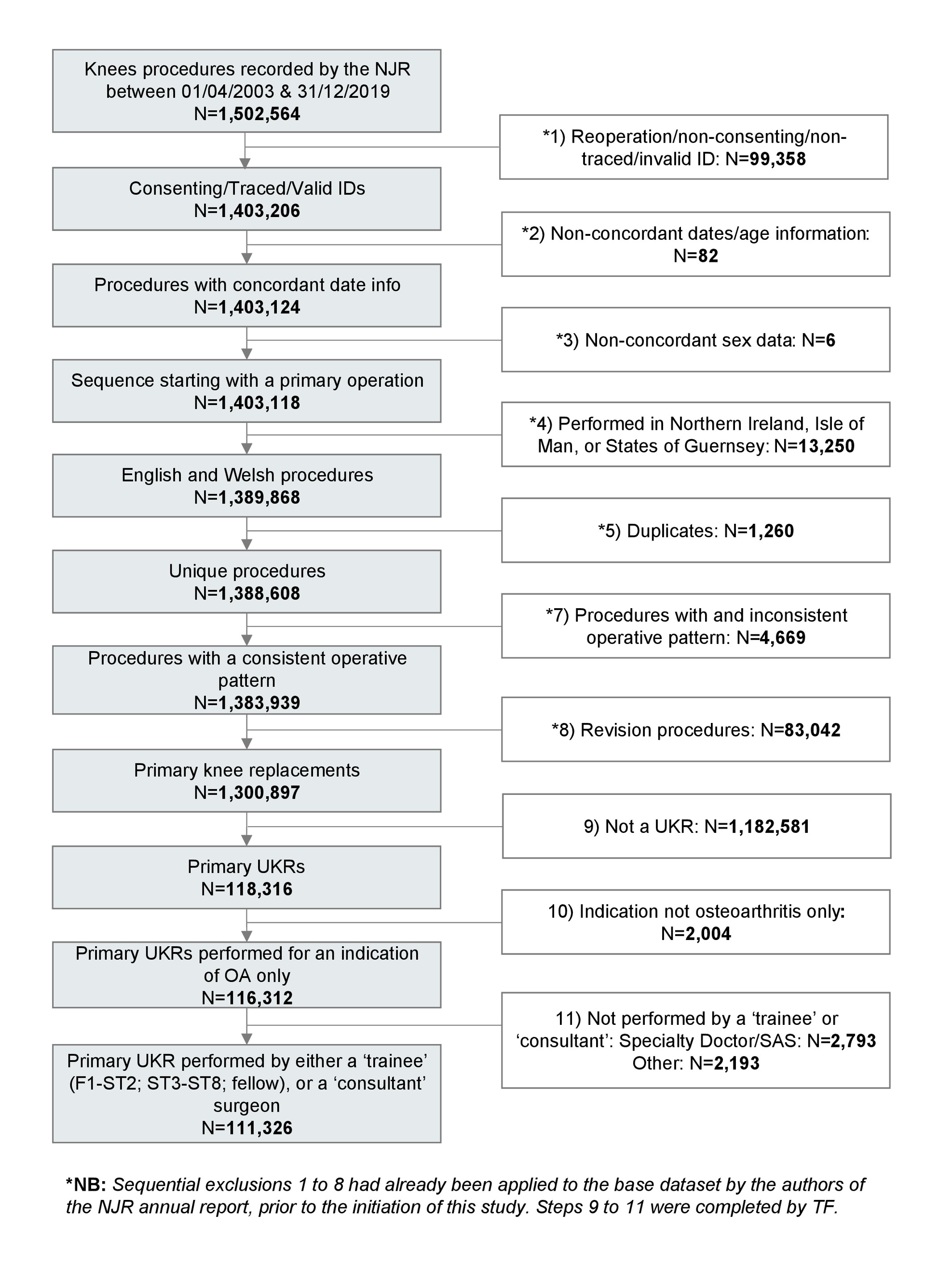

Supplement: S1 Fig — (TIF) [file pmed.1004445.s002.tif]

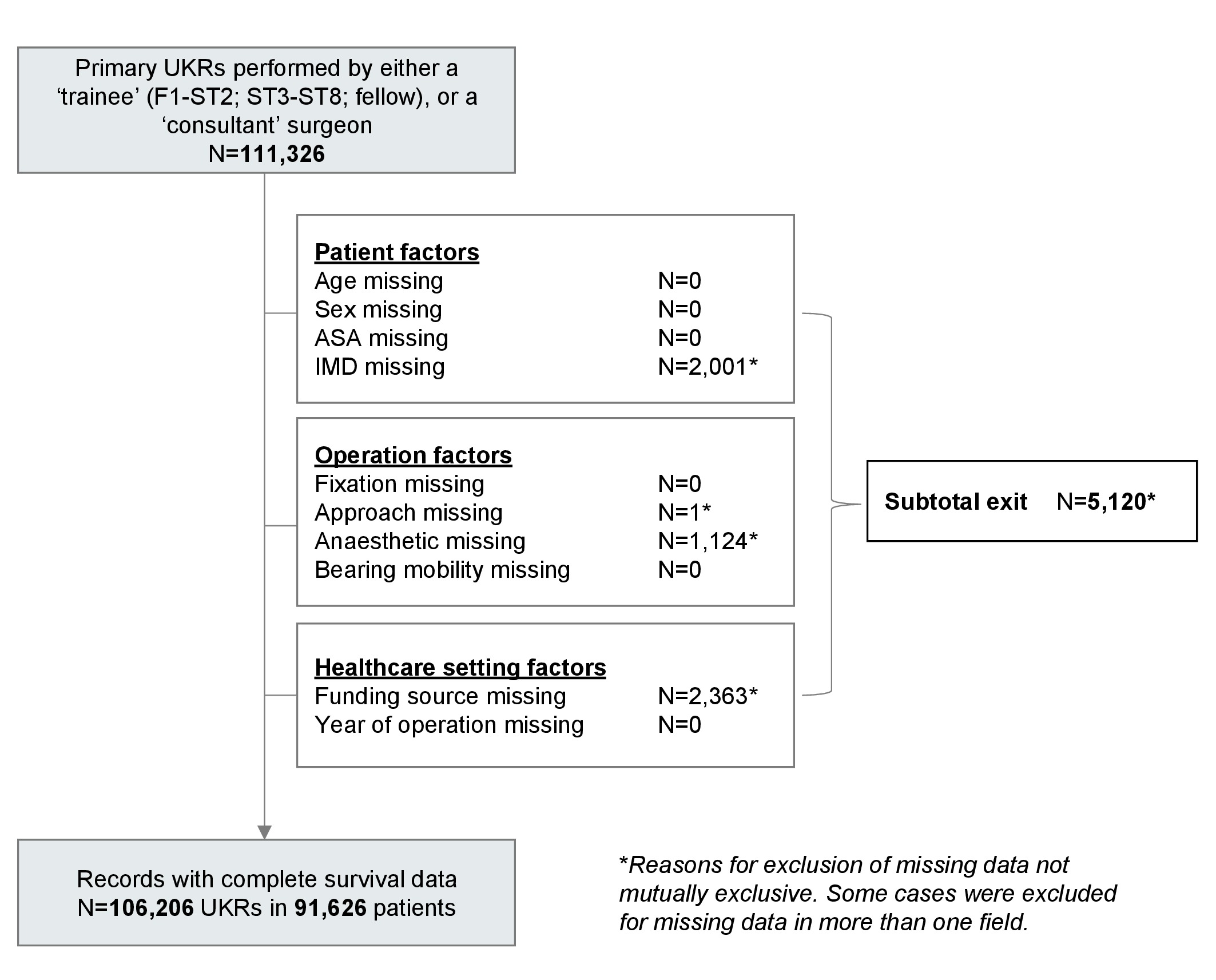

Supplement: S2 Fig — (TIF) [file pmed.1004445.s003.tif]
