# Supplementary material for: Association between surgeon training grade and the risk of revision following unicompartmental knee replacement: An analysis of National Joint Registry data
Source: PLoS Med. 2024 Sep 10;21(9):e1004445. doi: 10.1371/journal.pmed.1004445 (PMC11386457; doi:10.1371/journal.pmed.1004445)
Supplement: S2 Appendix — (DOCX) [file pmed.1004445.s005.docx]

S2 Appendix – Model selection, construction and justification

The structured approach to model selection and construction used in this study was based around an in-depth assessment of the proportionality of hazard functions. Where data did not satisfy the PH assumption (i.e. the ratio of hazard functions was not constant) and further analysis demonstrated that FPM was superior to the Cox model, FPM was preferred for primary analysis. This approach is summarised here:

**Step 1:** Schoenfeld residuals tests were applied to incrementally adjusted Cox models for ‘surgeon grade’ to assess the PH assumption.

1. Cox PH model for surgeon grade (Model 1: unadjusted)
   1. Schoenfeld residual test: p=0.143
2. Cox PH model for surgeon grade (Model 2: adjusted for patient factors)
   1. Schoenfeld residual test: p<0.001
3. Cox PH model for surgeon grade (Model 3: adjusted for patient & operation factors)
   1. Schoenfeld residual test: p<0.001
4. Cox PH model for surgeon grade (Model 4: adjusted for patient, operation & healthcare factors)
   1. Schoenfeld residual test: p<0.001

Schoenfeld residuals tests suggested that ‘surgeon grade’ did not have a significant time-dependent effect in this context. The lack of proportionality in adjusted Cox models was explained by the time-dependent effects of confounding variables. A range of methods were employed to investigate this further, including Schoenfeld residuals test/plots, and likelihood ratio testing comparing PH and non-PH models for each variable. We found evidence that the following variables had time-dependent effects: age; sex; IMD decile; approach; fixation; bearing mobility; funder and year of operation.

**Step 2:** The following FPMs were constructed to model the hazard function of ‘surgeon grade’:

1. A non-proportional hazards FPM (Model 4: adjusted for patient, operation & healthcare factors)
   1. A non-PH model using the stpm2 command in Stata, with the following variables specified as having a time-dependent effect: age, sex, IMD decile, approach, fixation, bearing mobility, funder, and year of operation.
   2. Graphical assessment, Akaike information criteria (AIC), and Bayes information criteria (BIC) were used to optimise the fit and complexity of the model. These methods were used to determine the degrees of freedom with which to model hazard functions, as well as the optimal number and location of knots.
   3. We confirmed the superiority of our final model to preceding iterations using likelihood ratio testing.
   4. The final model was as follows: The baseline hazard was modelled with 6 degrees of freedom (df). ‘Surgeon grade’ was modelled with fixed effects. The following variables were found to have time-dependent effects and the degrees of freedom used for each is denoted: age (1 df); sex (1 df); IMD decile (1 df); approach (1 df); fixation (3 df); bearing mobility (2 df); funder (3 df); year of operation (2 df). Remaining confounding variables were modelled with fixed effects.
2. A proportional hazards FPM (Model 4: adjusted for patient, operation & healthcare factors):
   1. A PH model using the stpm2 command in Stata, equivalent to the Cox model.
   2. The same as the non-PH FPM above, but with no time-dependent effects.

**Step 3:** Likelihood ratio tests were used to compare PH and non-PH models at each level of adjustment:

1. PH FPM compared to non-PH FPM (Model 1): N/A
2. PH FPM compared to non-PH FPM (Model 2): p<0.001
3. PH FPM compared to non-PH FPM (Model 3): p<0.001
4. PH FPM compared to non-PH FPM (Model 4): p<0.001

There was strong evidence to support the superiority of non-PH FPMs over PH models (i.e. Cox).

**Step 4**: Hazard ratio (HR) plots were produced using non-PH FPMs at each level of adjustment (Models 1-4) to graphically represent the hazard function for ‘surgeon grade’. The HR plots below show that there is no significant deflection of the HR above or below the baseline of one with any level of adjustment. Therefore, it was appropriate to quote numeric HRs rather than display HR plots.


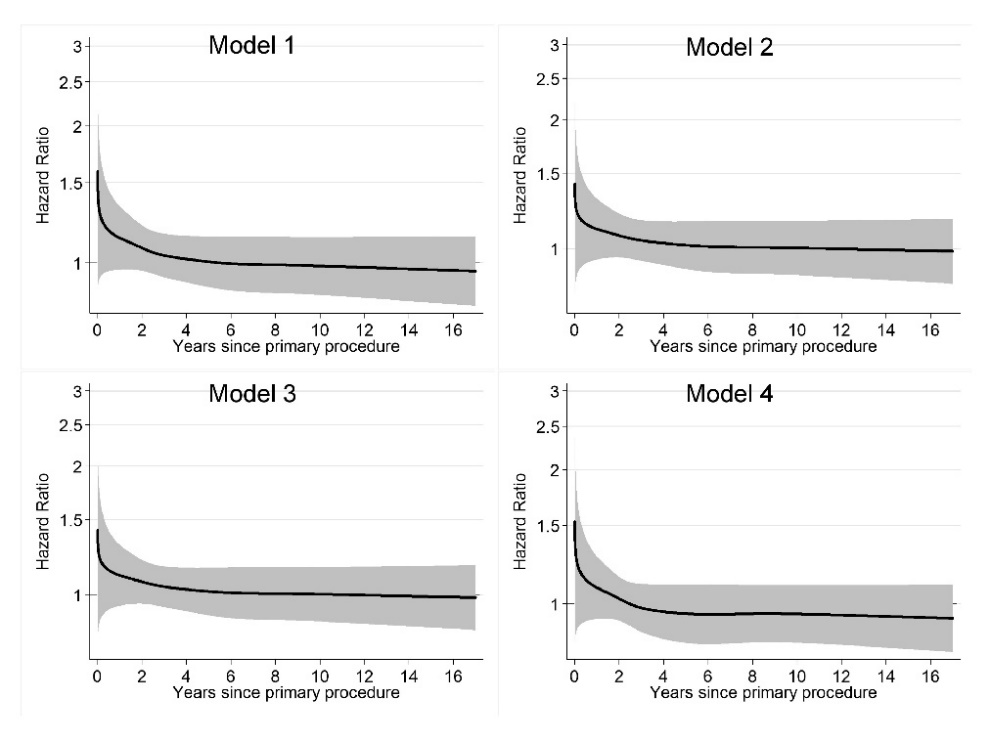
HR plots for non-PH FPMs at each level of adjustment. The risk of all cause revision of UKRs according to surgeon grade (exposure A).

**Summary:** We found strong evidence to support the superiority of non-PH FPMs over PH models (i.e. Cox) in this context. It was appropriate to present the results as numeric HRs.
