# Supplementary material for: Association between surgeon training grade and the risk of revision following unicompartmental knee replacement: An analysis of National Joint Registry data
Source: PLoS Med. 2024 Sep 10;21(9):e1004445. doi: 10.1371/journal.pmed.1004445 (PMC11386457; doi:10.1371/journal.pmed.1004445)

S3 Appendix – Schematic summary of surgical training in the UK.

*Schematic overview of surgical training in the UK. Adapted from: Fitzgerald JEF* *et al. (2012).*


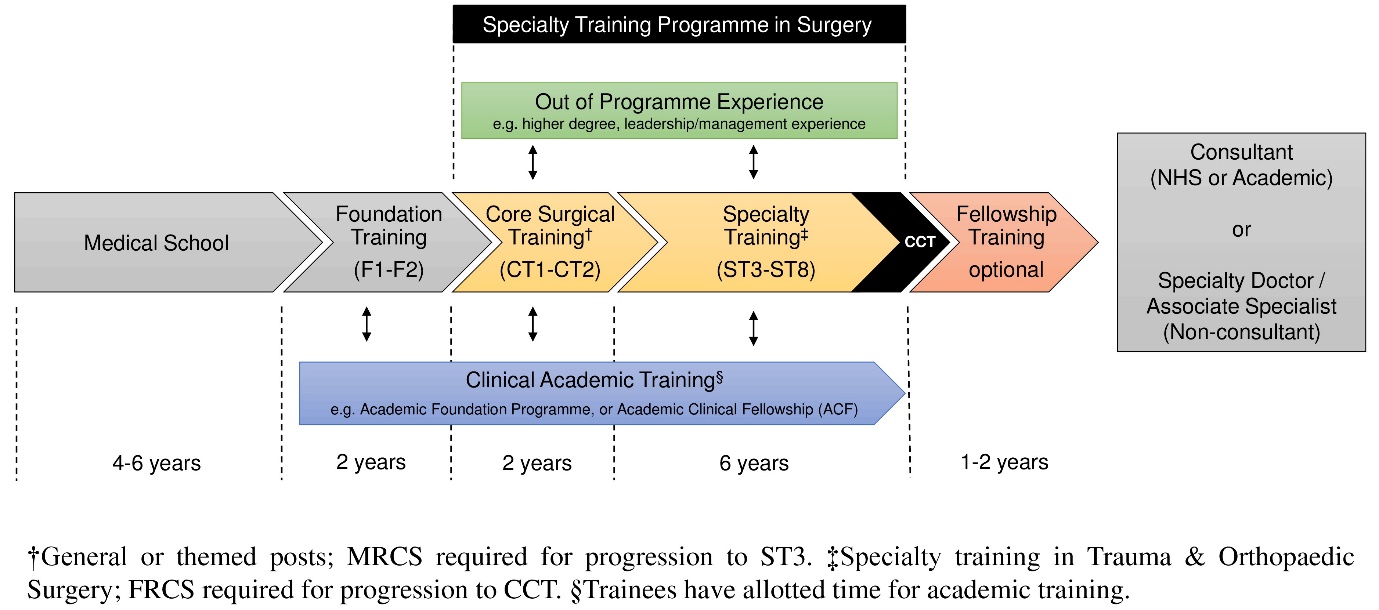

Supplement: S3 Appendix — (DOCX) [file pmed.1004445.s006.docx]
