# Supplementary material for: Association between surgeon training grade and the risk of revision following unicompartmental knee replacement: An analysis of National Joint Registry data
Source: PLoS Med. 2024 Sep 10;21(9):e1004445. doi: 10.1371/journal.pmed.1004445 (PMC11386457; doi:10.1371/journal.pmed.1004445)
Supplement: S4 Appendix — (DOCX) [file pmed.1004445.s007.docx]

S4 Appendix - Model specification summarising the exposures and confounding variables used in the analyses.

| **Model** | **Exposure (surgeon groups)** | **Method** | **Confounding variables included in model** |
| --- | --- | --- | --- |
| 1 | 1. **Surgeon grade:** i. Consultant; ii. Trainee (overall) | FPM | Unadjusted |
|  | 1. **Supervision:** i. Consultant; ii. Trainee supervised by a scrubbed consultant; iii. Trainee not supervised by a scrubbed consultant | FPM |  |
|  | 1. **Sensitivity analysis:** i. Consultant; ii. F1-ST2; iii. ST3-ST8; iv. Fellow (subcategorised according to consultant supervision) | FPM |  |
| 2 | 1. **Surgeon grade:** i. Consultant; ii. Trainee (overall) | FPM | Patient factors^†^ |
|  | 1. **Supervision:** i. Consultant; ii. Trainee supervised by a scrubbed consultant; iii. Trainee not supervised by a scrubbed consultant | FPM |  |
|  | 1. **Sensitivity analysis:** i. Consultant; ii. F1-ST2; iii. ST3-ST8; iv. Fellow (subcategorised according to consultant supervision) | FPM |  |
| 3 | 1. **Surgeon grade:** i. Consultant; ii. Trainee (overall) | FPM | Patient factors^†^  Operation factors^‡^ |
|  | 1. **Supervision:** i. Consultant; ii. Trainee supervised by a scrubbed consultant; iii. Trainee not supervised by a scrubbed consultant | FPM |  |
|  | 1. **Sensitivity analysis:** i. Consultant; ii. F1-ST2; iii. ST3-ST8; iv. Fellow (subcategorised according to consultant supervision) | FPM |  |
| 4 | 1. **Surgeon grade:** i. Consultant; ii. Trainee (overall) | FPM | Patient factors^†^  Operation factors^‡^  Healthcare setting factors^§^ |
|  | 1. **Supervision:** i. Consultant; ii. Trainee supervised by a scrubbed consultant; iii. Trainee not supervised by a scrubbed consultant | FPM |  |
|  | 1. **Sensitivity analysis:** i. Consultant; ii. F1-ST2; iii. ST3-ST8; iv. Fellow (subcategorised according to consultant supervision) | FPM |  |
| ^†^**Patient factors:** age (55-64yrs); gender (male); ASA (II); IMD decile (least deprived) | | | |
| ^‡^**Operation factors:** approach (medial parapatellar); mode of fixation (cemented); bearing mobility (mobile bearing); anaesthetic (spinal) | | | |
| ^§^**Healthcare setting factors:** funding (NHS); year of operation (most recent). ***Baseline category in brackets was the most frequently occurring*** | | | |
| **Time-dependent effects:** The following confounding variables were specified as having time-dependent effects: age (modelled with 1 degree of freedom (df)); sex (1 df); IMD decile (1 df); approach (1 df); fixation (3 df); bearing mobility (2 df); funder (3 df); year of operation (2 df). The baseline hazard was modelled with 6 df. Surgeon grade and the remaining confounding variables were modelled with fixed effects. | | | |
| ASA=American Society of Anaesthesiologists; NHS=National Health Service; IMD=Index of Multiple Deprivation; FPM=Flexible Parametric Survival Model; df=degrees of freedom; F1=Foundation Year 1; ST=Specialty Trainee (number denotes year of training). F1-ST2 is the most junior category, followed by ST3-ST8. | | | |
