# Supplementary material for: Association between surgeon training grade and the risk of revision following unicompartmental knee replacement: An analysis of National Joint Registry data
Source: PLoS Med. 2024 Sep 10;21(9):e1004445. doi: 10.1371/journal.pmed.1004445 (PMC11386457; doi:10.1371/journal.pmed.1004445)
Supplement: Attachment — Submitted filename: fowler.pdf [file pmed.1004445.s008.pdf]

This paper uses registry data to examine the association between surgeon grade and patient outcomes.

I was asked for a methodological report and I interpret that to include all aspects of the design and conduct of the study. The version I received did not have page numbers. My numbering has the abstract starting on page 2

Points of detail

Page 7 Technically speaking the amount of missingness is part of the results not the methods but it can be difficult to maintain the distinction.

Page 8 I think for an international readership more detail is needed about the various stages in the UK medical career. Is it the case, for example, that after passing through ST8 trainees automatically become consultants? Can they skip stages? One possible piece of information which might help map this on to other countries is the length of time typically spent in each stage. I think I can work out what scrubbed means but again that might be worth explaining.

Page 9 In fact the authors appear to have divided IMD into five categories not ten as it appears here. Or is that just for Table 1?

Page 11 This might be the appropriate place to report and comment on the amount of missingness and justify the decision to use complete cases. As I see it there is about 5% missing which would make the need for some form of imputation marginal. Was the decision not to use BMI (which did have substantial missingness) taken before or after establishing the amount of missingness?

Page 13 Would we have expected progression of OA to be associated with the skill level of the surgical team? Or was this included as a possible negative control?

Page 18 I had to re–read the sentence starting ‘However, as they are’ until the penny dropped since I was looking back for the plural noun to which they referred. Perhaps I was being unusually dense but I would suggest moving the explanation (from ‘as they’ to ‘NJR’) to the end after what it explains

Page 19 I am not sure that OA ‘accounts’ for case–mix. I can see that it reduces variability to focus on one indication. Perhaps account means something different for me.

Figure 2 I am afraid that the presentation of the confidence intervals using

two colours has not been a huge success. When overprinted they all look pink to me. What happens if the authors just plot the limits of the regions as lines in the appropriate colour? Or increase the saturation of the consultant band?

Checklist I wonder whether, since this uses routinely collected data, the RECORD guideline might be more appropriate (Benchimol et al., 2015)?

Points of more substance

Factors driving allocation

The key limitation which the authors point out is that we do not know why some operations are carried out by consultants and some not. It would be helpful if the authors can confirm that there are no further steps they can take to adjust for any factors which might have influenced the decision. This is not a fatal problem with the paper since it is hard to envisage a realistic study design to avoid the problem

Both knees operated

I notice from Figure 1 that 14000+ people had both knees operated on. Strictly speaking this introduces a lack of independence between observations because they are on the same person. The usual solution would be to add a frailty term.

Perhaps more interestingly if the operations are not carried out simultaneously there may be scope for a different analysis. If there are sufficient pairs of knees which were operated on by surgeons of different levels then a within–person analysis would be possible. This would hold patient characteristics more or less constant and probably some of the other covariates too. I know nothing about surgical practice in this area and it may be that both knees are always done at the same time.

Summary

Some points for clarification and a couple of issues with the analysis.

Michael Dewey

References

E I Benchimol, L Smeeth, A Guttmann, K Harron, D Moher, I Petersen, H Sørensen, E von Elm, S Langan, and RECORD working committee. The REporting of studies Conducted using Observational Routinely–collected health Data(RECORD) Statement. *PLOS Medicine*, 12(10), 2015.
