# Supplementary material for: Association between surgeon training grade and the risk of revision following unicompartmental knee replacement: An analysis of National Joint Registry data
Source: PLoS Med. 2024 Sep 10;21(9):e1004445. doi: 10.1371/journal.pmed.1004445 (PMC11386457; doi:10.1371/journal.pmed.1004445)
Supplement: Attachment — Submitted filename: Response.docx [file pmed.1004445.s009.docx]

Mr T J Fowler

Musculoskeletal Research Unit

Translational Health Sciences

Bristol Medical School

Southmead Hospital, Bristol

United Kingdom

**Prof. Dr. Till Bärnighausen**

Editor-in-Chief

PLOS Medicine

May 11th, 2024

Dear **Prof. Dr. Bärnighausen**,

**Reply to comments: “Association between surgeon training grade and the risk of revision following unicompartmental knee replacement: an analysis of a National Joint Registry Data” (PMEDICINE-D-24-00668R1)**

Thank you for taking the time to review our study and for the detailed appraisal of our work. We would like to thank the individual reviewers for their time and constructive comments. I am pleased to attach our revised article, as well as our detailed response to each of the reviewers’ comments. We have attempted to address all comments and have edited the manuscript accordingly. We feel that the changes that we have made in response to reviewer’s comments have improved the quality of the manuscript as a whole.

Yours sincerely,

Mr Tim Fowler

BM BCh (Oxon) MA FRCS

Academic Clinical Fellow & Orthopaedic Registrar

Musculoskeletal Research Unit, Bristol Medical School, Southmead Hospital, Bristol, UK

Email: t.j.fowler@bristol.ac.uk; Phone: +44 (0)117 414 7865

| **Reply to comments: “Association between surgeon training grade and the risk of revision following unicompartmental knee replacement: an analysis of a National Joint Registry Data” (PMEDICINE-D-24-00668R1)** | | |
| --- | --- | --- |
|  | | |
| **Reviewer & Comment number** | **Comment** | **Response** |
| **Reviewer 1: Comment 1** | *Generally the methodology is satisfactory within the framework of using registry data. The authors do not distinguish between lateral and medial UKR which I find a little unusual when we know that lat UKR especially with a mobile bearing are technically more difficult with a higher revision rate. It would be interesting to know if there is indeed a difference in the UK. If the authors cannot easily separate the med from lat UKRs then I would like to see a section relating to this in the limitation section.* | We are very grateful for the time you have taken to review our work and for your constructive comments. We agree that the distinction between medial and lateral unicompartmental is an important one and would have liked to make this distinction in our analyses had the information been available to us.  The laterality (medial or lateral) of unicompartmental knee replacements is not yet reported by the NJR and thus this information was not available to us. The NJR explain this in their most recent 20^th^ Annual Report (p145): *“We note here that the NJR data collection process now distinguishes between medial and lateral unicondylar replacements, although this was not always the case in the past. This distinction is available for cases reported on the MDSv7 forms, but unicondylar cases reported on earlier versions of the MDS form do not make this distinction. Work is ongoing to determine if data entered in previous versions of the MDS forms can be used to identify medial and lateral replacements. If this is possible, it will be reported in future annual reports.”*  This information was not recorded by the NJR until 2018 at the earliest, and not routinely recorded until 2019. The dataset we had access to did not include this information. This is also the case for previous publications based on NJR UKR data [1, 2]:  <https://doi.org/10.1016/S0140-6736(14)60419-0>  <https://doi.org/10.1016/j.arth.2020.08.063>  As per your recommendation, we have now added a discussion of this to the limitation section:  “The distinction between medial and lateral UKRs is not routinely reported by the NJR. The NJR data collection process did not distinguish between medial and lateral UKRs until the introduction of MDS version 7 in 2018 and this information was not available within the dataset.” |
| **Reviewer 1: Comment 2** | *Progression of OA was the commonest cause for revision and I would like the authors to expand on the implications of this cause for revision within the framework of the study - progression of OA is hardly a failure of the implant and more likely to be due to inappropriate patient selection (overstuffing may have occurred but is probably much less likely to cause progression of OA especially as we know that there continues to be subsidence of both cemented and uncemented implants over time), not being aware of OA elsewhere at the time of surgery, which is likely to be a decision event which is outside of the trainee's brief. Although the actual revision rate for progression of OA is similar across all surgeons a comment on this would be useful.* | Revision is defined by the NJR as “any operation where one or more components are added to, removed from, or modified in a joint replacement [3].” Therefore, changing the implant construct in response to a progression of arthritis in previously unreplaced compartments is recorded by the NJR as an episode of revision. This might include revising a UKR to a TKR, or the addition of a lateral UKR to a knee which has previously had a medial UKR.  We agree that this does not necessarily reflect a failure of the implant components themselves. But in view of the NJR definition of revision, this is recorded by the NJR as a revision event/episode of failure. As you correctly point out, there was no evidence of an association between surgeon grade/supervision and the risk of revision for progressive arthritis. We agree that the risk of revision for this indication may be more likely to relate to appropriate patient selection rather than failure of the implant components themselves. It is also likely that the patients have been selected by the responsible consultant, rather that the trainee. However, it is not possible to determine this from the data.  In response to your recommendation, we have added a comment on this topic to the discussion section:  “The most common indication for revision was progression of OA. The NJR defines revision as any procedure to add, remove, or modify one or more components of an implant construct for any reason [3]. Revision for progression of OA in the context of previous UKR implies progression of arthritis in previously unreplaced compartments of the knee. This includes procedures such as revising the UKR to a TKR, or the addition of another UKR (medial, lateral, or patellofemoral) to a previously unreplaced compartment. It does not necessarily imply failure of the individual implant components but is recorded by the NJR as a failure of the construct. We found no evidence of an association between surgeon grade, or the level of supervision and the risk of revision for progression of OA, which suggests that trainers are selecting appropriate cases for their trainees.” |
| **Reviewer 1: Comment 3** | *The authors comment on the limitation of no PROMs data which is a significant limitation, especially when considering unexplained pain, however within the confines of registry data this is acceptable. Otherwise I believe the authors should be congratulated for a robust and sound study.* | Thank you for your feedback. We agree that the lack of PROMs data is a notable limitation and have commented on this in the limitations section. |
| **Reviewer 2 (Michael Dewey): Comment 1** | The version the reviewer received did not have page numbers. Their numbering has the abstract starting on page 2.  *Page 7: Technically speaking the amount of missingness is part of the results* *not the methods but it can be difficult to maintain the distinction.* | We are very grateful for the time you have taken to review our work and for your constructive comments. Page and line numbers have now been added to the document. The numbering still has the abstract starting on page 2.  Thank you for your recommendation. We have now moved the comment on the missingness of data and justification for complete case analysis to the results section. |
| **Reviewer 2: Comment 2** | *Page 8: I think for an international readership more detail is needed about the various stages in the UK medical career. Is it the case, for example, that after passing through ST8 trainees automatically become consultants? Can they skip stages? One possible piece of information which might help map this on to other countries is the length of time typically spent in each stage. I think I can work out what scrubbed means but again that might be worth explaining.* | This is a very good point. Therefore, we have added the following detailed explanation of UK orthopaedic training:  “F1-ST2 represents the first four years of postgraduate training after graduating from medical school (F1, F2, ST1 and ST2). ST2 doctors who have completed the Membership of the Royal College of Surgeons (MRCS) examination are eligible to apply to Specialty Training. Specialty Training in Trauma and Orthopaedic Surgery is typically a six-year programme (ST3-8). ST3-ST8 trainees are referred to as ‘registrars’ Progression through training levels is dependent on the successful completion of training requirements and competencies. Trainees who have completed ST6 are eligible to sit the examination for Fellowship of the Royal College of Surgeons (FRCS), which is mandatory for Certification of Completion of Training (CCT). Trainees progress to post-CCT fellowship training prior to applying for a consultant position. The term ‘consultant’ is synonymous with ‘attending’, and the term ‘registrar’ is synonymous with ‘resident’ in many healthcare settings including the USA. A schematic summary of the stages of surgical training in the UK is included in Supplementary Methods 2 [4].” |
| **Reviewer 2: Comment 3** | *Page 9: In fact the authors appear to have divided IMD into five categories not ten as it appears here. Or is that just for Table 1?* | IMD deciles were used for analysis. The deciles are summarised as five categories in Table 1 in attempt to keep it tidy and prevent splitting the table over multiple pages. We have added a * to the table footnote to clarify this. |
| **Reviewer 2: Comment 4** | *Page 11:*  *This might be the appropriate place to report and comment on the amount of missingness and justify the decision to use complete cases. As I see it there is about 5% missing which would make the need for some form of imputation marginal.*  *Was the decision not to use BMI (which did have substantial missingness) taken before or after establishing the amount of missingness?* | Thank you for your recommendation. We have now moved the comment on the missingness of data and justification for complete case analysis to the results section. This reads as follows:  “Details of missing data are documented in Supplementary Figure ii. Fewer than 5% of cases (n=5,120) had missing data. Complete-case analysis was used in all analyses and records with missing data in any confounding variable field used in subsequent statistical models were excluded from the relevant model. This is based on the assumption that the pattern of missingness of NJR data is independent of the primary exposure and the outcome. Considering the large datasets, the small proportion of incomplete cases, and the assumed pattern of missingness, any potential improvement in efficiency from using multiple imputation compared to complete-case analysis is likely to be negligible [5, 6].”  We have added the following text to the methods section (statistical analysis sub section):  “Body mass index (BMI) is missing in a large proportion of NJR records. It has been reported that approximately 40% of patients did not have a BMI recorded in the NJR in 2009, compared to approximately 18% in 2016 [7]. Due to the significant proportion of records with missing values, BMI was not included as a confounding variable in the analyses. This is consistent with the approach used in previous NJR studies and this decision was made prior to initiating the study based on the known pattern of missing data [5, 8].” |
| **Reviewer 2: Comment 5** | *Page 13: Would we have expected progression of OA to be associated with the skill level of the surgical team? Or was this included as a possible negative control?* | Progression of OA as an indication for revision was also raised by Reviewer 1. I have included my previous response below:  Revision is defined by the NJR as “any operation where one or more components are added to, removed from, or modified in a joint replacement [3].” Therefore, changing the implant construct in response to a progression of arthritis in previously unreplaced compartments is recorded by the NJR as an episode of revision. This might include revising a UKR to a TKR, or the addition of a lateral UKR to a knee which has previously had a medial UKR.  We agree that this does not necessarily reflect a failure of the implant components themselves. But in view of the NJR definition of revision, this is recorded by the NJR as a revision event/episode of failure. As you correctly point out, there was no evidence of an association between surgeon grade/supervision and the risk of revision for progressive arthritis. We agree that the risk of revision for this indication may be more likely to relate to appropriate patient selection rather than failure of the implant components themselves. It is also likely that the patients have been selected by the responsible consultant, rather that the trainee. However, it is not possible to determine this from the data.  In response to your recommendation, we have added a comment on this topic to the discussion section:  “The most common indication for revision was progression of OA. The NJR defines revision as any procedure to add, remove, or modify one or more components of an implant construct for any reason [3]. Revision for progression of OA in the context of previous UKR implies progression of arthritis in previously unreplaced compartments of the knee. This includes procedures such as revising the UKR to a TKR, or the addition of another UKR (medial, lateral, or patellofemoral) to a previously unreplaced compartment. It does not necessarily imply failure of the individual implant components but is recorded by the NJR as a failure of the construct. We found no evidence of an association between surgeon grade, or the level of supervision and the risk of revision for progression of OA, which suggests that trainers are selecting appropriate cases for their trainees.” |
| **Reviewer 2: Comment 6** | *Page 18: I had to re-read the sentence starting `However, as they are' until the penny dropped since I was looking back for the plural noun to which they referred. Perhaps I was being unusually dense, but I would suggest moving the explanation (from `as they' to `NJR') to the end after what it explains.* | We agree with this point and have edited the sentence according to your recommendation. |
| **Reviewer 2: Comment 7** | *Page 19: I am not sure that OA `accounts' for case-mix. I can see that it reduces variability to focus on one indication. Perhaps account means something different for me.* | This is a fair point. We have removed the term ‘case-mix selection’ from this sentence. |
| **Reviewer 2: Comment 8** | *Figure 2: I am afraid that the presentation of the confidence intervals using two colours has not been a huge success. When overprinted they all look pink to me. What happens if the authors just plot the limits of the regions as lines in the appropriate colour? Or increase the saturation of the consultant band?* | We have edited Figure 2 to increase the saturation of the consultant band according to your recommendation. |
| **Reviewer 2: Comment 9** | Checklist I wonder whether, since this uses routinely collected data, the RECORD guideline might be more appropriate (Benchimol et al., 2015)? | Thank you for this recommendation. We have now updated our checklist to use the RECORD checklist. We have included a citation for this in the methods section.  To comply with the RECORD guidelines, we have included additional information on the data linkage that is carried out by the NJR, prior to us obtaining the dataset. We have included the following statement in the ‘Data processing’ subsection of the methods:  “NJR data are annually linked to other healthcare system datasets, including Civil Registration Authority data, using unique patient identifiers. This linkage, which was carried out by the NJR prior to us obtaining the dataset, is approved by the Health Research Authority under Section 251 of the NHS act 2006 [3].” |
| **Reviewer 2: Comment 10** | *Factors driving allocation: The key limitation which the authors point out is that we do not know why some operations are carried out by consultants and some not. It would be helpful if the authors can confirm that there are no further steps they can take to adjust for any factors which might have influenced the decision. This is not a fatal problem with the paper since it is hard to envisage a realistic study design to avoid the problem.* | This is another excellent point, and we agree that this is a limitation of this observational study, which is based on registry data. We agree that there is likely to be a non-random allocation of cases by consultants to trainees. We have attempted to account for this by incrementally adjusting for a comprehensive range of patient, operation, and healthcare setting factors (including: Age, sex, ASA grade, IMD, mode of anaesthesia, surgical approach, fixation of implants, bearing mobility, funding source and year of operation). We acknowledge that there may be residual confounding and can confirm that there are no further steps that we can take to adjust for any factors that might have influenced allocation of cases to trainees. Whilst this makes it difficult to fully understand the true training effect, our results suggest that current training practices in Egland and Wales, including the process of allocation of appropriate cases to trainees, are safe and effective for patients.  To reiterate this point we have added the following to the limitations section:  “This is an observational study and there is likely to be a non-random allocation of cases by consultants to trainees. We have attempted to account for this by adjusting for a comprehensive range of confounding variables. However, we acknowledge that there may be residual confounding and confirm that, to our knowledge, there are no further steps to take to adjust for factors that might have influenced the allocation of cases. While this may make it difficult to understand what the true training effect is, our results suggest that the current process of allocating UKR cases to trainees in England and Wales is safe and effective.” |
| **Reviewer 2: Comment 11** | *Both knees operated: I notice from Figure 1 that 14000+ people had both knees operated on. Strictly speaking this introduces a lack of independence between observations because they are on the same person. The usual solution would be to add a frailty term. Perhaps more interestingly if the operations are not carried out simultaneously there may be scope for a different analysis. If there are sufficient pairs of knees which were operated on by surgeons of different levels, then a within person analysis would be possible. This would hold patient characteristics more or less constant and probably some of the other covariates too. I know nothing about surgical practice in this area and it may be that both knees are always done at the same time.* | This is a very interesting point and one which we had not considered. With regards to your first point, we have addressed this with the following additional analysis:  **Last paragraph of statistical methods section:**  “We conducted an additional sensitivity analysis to explore the lack of independence between observations in patients who underwent bilateral procedures (on the same day, or on different days). We examined the primary outcome measure using a multilevel mixed effects parametric survival model to introduce a frailty term and account for time-dependent effects [9].”  **Last paragraph of ‘All-cause revision’ subsection of methods:**  “In response to the peer review process, an additional sensitivity analysis was performed to explore the lack of independence between observations in patients who underwent bilateral procedures. The results were very similar, and we found no evidence of an association between surgeon grade (Model 4: HR = 1.01, 95% CI [0.90, 1.13]; p*=*0.89) and the risk of all-cause revision.”  To do this we used the *stmixed* command in Stata and specified bilateral cases. We have cited the appropriate paper. The results were very similar (almost identical). We also used an alternative approach in which we excluded the second bilateral procedure (same day, or different day). Again, the results were almost identical. For simplicity we have not included this, but it is reassuring.  With regards to your second point. This is another excellent point. We have identified 703 pairs of knees, which were replaced on different days by surgeons of different grades (consultant vs. trainee). There are <80 revision events in total amongst these pairs. This would be a very interesting further analysis. However, we feel that the number of cases and events is likely to be too small for a meaningful analysis at present. This would however be reasonable to look at in future studies with bigger numbers, e.g. in the context of total hip replacements, or total knee replacements (over a million of each in the NJR). |
| **Reviewer 3:**  **Comment 1** | *The authors evaluated patient outcomes following unicompartmental knee joint replacement surgery upon the variable of whether the surgeon was a consultant or trainee and demonstrated that there was no difference in the primary outcome - need for revision surgery with long term follow up.  This is a national registry study and is subject to limitations which the authors transparently discussed.  Given that this is the most common method of training surgical trainees, I think this is a valuable contribution to the literature and provides insight into potential risk or lack thereof with our current methods.* | Thank you for your positive feedback. We are very grateful for the time you have taken to review our work and for your constructive comments. No specific comments to address. |
| **Reviewer 4:**  **Comment 1** | *The authors investigated the revision rate of UKA in the NJR, by controlling for trainee vs consultants. Please add line numbers to facilitate review.* | We are very grateful for the time you have taken to review our work and for your constructive comments. Page and line numbers have now been added to the main manuscript document. |
| **Reviewer 4:**  **Comment 2** | *The main issue is controlling for consultant surgeon, in light of the data on UKA. There is sufficient evidence demonstrating that UKA should be performed on a regular basis to decrease revision. Thus, comparing low volume consultant surgeons with trainees might omit the true data. In these papers (10.1007/s00167-021-06650-4), the cut-off of consultant with at least 100 knee arthroplasty was used, which is a more fair comparison. I suggest adding this analysis. The paper is otherwise well written.* | This is an excellent point. The term ‘surgeon volume’, which is often used interchangeably with ‘surgeon caseload’, is typically used to describe the number of operations performed by an individual surgeon in a calendar year. In the arthroplasty literature, it has been used to describe a surgeon’s mean annual volume [10, 11], or a surgeon’s volume in the 365 days preceding an index procedure [5, 12, 13]. Researchers have used several different methodological approaches to investigate the association between surgeon volume and the risk of revision following joint replacement [5, 10, 13, 14].  Surgeon volume is a valuable, but distinct measure of surgical experience compared to the binary variable ‘surgeon grade’, which we have used in this study. We acknowledge this point in our introduction (Introduction: Paragraph 1) and make further reference to this term in our discussion (Discussion: Paragraph 1). NJR studies have identified that UKRs are susceptible to a low-volume effect, whereby low surgeon volume is associated with significantly higher revision rates [10, 11, 14]. However, surgeon volume is a very different topic to the one discussed in the current paper.  The New Zealand Joint Registry Study that you have referenced refers to the ‘usage’ of UKRs [15]. This is another term, which describes the proportion of a surgeon’s knee replacement practice (UKR + TKR) that includes UKRs. This is another interesting topic but is distinct from ‘surgeon volume’ or ‘surgeon grade’. The topic of usage has also been looked at in the NJR [16].  If we were to conduct an additional analysis using categories for low-, or high-volume consultant surgeons then it might be more appropriate to use the evidenced-based UKR volume categories, which were described by Liddle et al. within the context of NJR data (low-volume (<10 cases/year), medium-volume (≥10 & <30 cases/year), and high-volume (≥30 cases/year) [10].  However, please look at our group’s previous work on the topic of surgeon volume in the context of joint replacement surgery/NJR analysis [5, 17]. Consultant surgeon volume changes dynamically over time. Thus, we feel that using arbitrary volume categories is a significant oversimplification, which we are keen to avoid.  To include an additional sensitivity analysis using ‘surgeon volume’ as a variable, we would need to account for annual variations in consultnat surgical volume and use a multilevel mixed effects parametric (Weibull) survival model. As you can see from our previous work on this subject, this would be a significicant undertaking. We appreciate your comment and agree that this would be a very interesting topic for further investigation. However, we feel that this would overcomplicate the current paper, would detract from the current take home message, and would be best suited to a separate study. |
| **General Editorial Request: 1** | *Please upload any figures associated with your paper as individual TIF or EPS files with 300dpi resolution at resubmission; please read our figure guidelines for more information on our requirements:* [*http://journals.plos.org/plosmedicine/s/figures*](http://journals.plos.org/plosmedicine/s/figures)*. While revising your submission, please upload your figure files to the PACE digital diagnostic tool, https://pacev2.apexcovantage.com/. PACE helps ensure that figures meet PLOS requirements. To use PACE, you must first register as a user. Then, login and navigate to the UPLOAD tab, where you will find detailed instructions on how to use the tool. If you encounter any issues or have any questions when using PACE, please email us at PLOSMedicine@plos.org.* | This has been addressed. |
| **General Editorial Request: 2** | *Please ensure that the paper adheres to the PLOS Data Availability Policy (see http://journals.plos.org/plosmedicine/s/data-availability), which requires that all data underlying the study's findings be provided in a repository or as Supporting Information. For data residing with a third party, authors are required to provide instructions with contact information for obtaining the data. PLOS journals do not allow statements supported by "data not shown" or "unpublished results." For such statements, authors must provide supporting data or cite public sources that include it.* | Please see our updated data availability statement, which reads as follows: “The data used in the study are available from The National Joint Registry (NJR) (https://www.njrcentre.org.uk). Restrictions apply to the availability of these data, which were used under license for the current study, and are therefore not publicly available. Data access applications can be made to the NJR Research Committee. With NJR permission in place, the data underlying the presented results will be available to access via the NJR data access network. The authors of this manuscript are not the data owner and do not have permission to share the data.” |
| **General Editorial Request: 3** | *We ask every co-author listed on the manuscript to fill in a contributing author statement, making sure to declare all competing interests. If any of the co-authors have not filled in the statement, we will remind them to do so when the paper is revised. If all statements are not completed in a timely fashion this could hold up the re-review process. If new competing interests are declared later in the revision process, this may also hold up the submission. Should there be a problem getting one of your co-authors to fill in a statement we will be in contact. YOU MUST NOT ADD OR REMOVE AUTHORS UNLESS YOU HAVE ALERTED THE EDITOR HANDLING THE MANUSCRIPT TO THE CHANGE AND THEY SPECIFICALLY HAVE AGREED TO IT. You can see our competing interests policy here: http://journals.plos.org/plosmedicine/s/competing-interests.* | Please see our competing interests statement. Please let me know if further information is required.  “We have read the journal’s policy and the authors of this manuscript have the following competing interests: AWB and MRW declare support from The Healthcare Quality Improvement Partnership/The NJR in the form of the Lot 2 contract for statistical analysis of the NJR, during the conduct of the submitted work; AWB and MRW report grants from the NIHR investigating the outcomes of joint replacement, outside the submitted work; AWB and MRW are editors of an Orthopaedic textbook for which they receive royalty payments from Taylor Francis; MRW reports fees paid to their institution for delivering teaching at courses organised by DePuy and Heraeus; no other relationships or activities that could appear to have influenced the submitted work.” |
| **General Editorial Request: 4** | *Data Availability - thank you for including a statement regarding data availability. As the data are not freely available, please include an appropriate contact (web or email address) for inquiries to the National Joint Registry. Please note that this cannot be a study author.* | Please see our updated data availability statement, which reads as follows: “The data used in the study are available from The National Joint Registry (NJR) (https://www.njrcentre.org.uk). Restrictions apply to the availability of these data, which were used under license for the current study, and are therefore not publicly available. Data access applications can be made to the NJR Research Committee. With NJR permission in place, the data underlying the presented results will be available to access via the NJR data access network. The authors of this manuscript are not the data owner and do not have permission to share the data.” |
| **General Editorial Request: 5** | *Please ensure that the study is reported according to the RECORD (STROBE if you feel it is more appropriate) guideline and include the completed RECORD checklist as Supporting Information. Please add the following statement, or similar, to the Methods: "This study is reported as per the Reporting of studies Conducted using Observational Routinely-collected health Data (RECORD) Statement (S1 Checklist)." When completing the checklist, please use section and paragraph numbers, rather than page numbers.* | We have now replaced the STROBE checklist with the RECORD checklist according to your recommendation. This is cited within the methods section and the checklist has been uploaded with the submission.  Please see our response to Reviewer 2 (Comment 9). Additional information has been added to the methods section to meet the requirements of the RECORD checklist. |
| **General Editorial Request: 6** | *Statistical reporting: Throughout, including the abstract, please quantify the main results with 95% CIs and p values. When reporting p values, please report as <0.001 and where higher as p=0.002, for example. When reporting 95% CIs, please separate upper and lower bounds with commas instead of hyphens as the latter can be confused with reporting of negative values. Please include the actual amounts and/or absolute risk(s) of relevant outcomes (including NNT or NNH where appropriate), not just relative risks or correlation coefficients. (example for absolute risks: PMID: 28399126). Please include any important dependent variables that are adjusted for in the analyses.* | We have reviewed the manuscript and made changes were necessary. This has now been addressed. |
| **General Editorial Request: 7** | *We appreciate that this is a retrospective study, but did the study have a prospective protocol or analysis plan? Please state this (either way) early in the Methods section.*  *a) If a prospective analysis plan (from your funding proposal, IRB or other ethics committee submission, study protocol, or other planning document written before analyzing the data) was used in designing the study, please include the relevant prospectively written document with your revised manuscript as a Supporting Information file to be published alongside your study, and cite it in the Methods section. A legend for this file should be included at the end of your manuscript.*  *b) If no such document exists, please make sure that the Methods section transparently describes when analyses were planned, and when/why any data-driven changes to analyses took place.*   *c) In either case, changes in the analysis-- including those made in response to peer review comments-- should be identified as such in the Methods section of the paper, with rationale.*  *For all observational studies, in the manuscript text, please indicate: (1) the specific hypotheses you intended to test, (2) the analytical methods by which you planned to test them, (3) the analyses you actually performed, and (4) when reported analyses differ from those that were planned, transparent explanations for differences that affect the reliability of the study's results. If a reported analysis was performed based on an interesting but unanticipated pattern in the data, please be clear that the analysis was data-driven.* | The study protocol was planned in detail prior to commencing the study, including defining the study population, exposures, outcomes of interest and statistical methods. The main analyses were planned prior to commencing the study and these are documented throughout the methods section. However, data-driven changes to the analysis took place and these are summarised in detail in Supplementary Methods 3, which justifies the model selection and construction.  An additional sensitivity analysis has been added according to the recommendation of Reviewer 2 (See Reviewer 2: Comment 11). We have outlined in the methods section that this was added in response to the peer review process.  The study protocol was not published prior to commencing the study. Thus, no such document exists. We have clarified this early in the methods section according to your request. The data-driven changes to the analysis outlined in Supplementary Methods 3 are also referenced in the methods section.  We have added the following paragraph to the second paragraph of the methods section:  “**Analysis plan**  The study protocol was designed prior to commencing the study, including defining the study population, exposures, outcomes of interest and statistical methods. The main analyses were planned prior to commencing the study and these are documented throughout this methods section. However, data-driven changes to the analysis took place and these are summarised in detail in Supplementary Methods 2, which justifies the model selection and construction.” |
| **General Editorial Request: 8** | *Abstract - please structure your abstract using the PLOS Medicine headings (Background, Methods and Findings, Conclusions).* | This has been addressed. |
| **General Editorial Request: 9** | *Author summary - at this stage, we ask that you include a short, non-technical Author Summary of your research to make findings accessible to a wide audience that includes both scientists and non-scientists. The authors summary should consist of 2-3 succinct bullet points under each of the following headings: • Why Was This Study Done? Authors should reflect on what was known about the topic before the research was published and why the research was needed. • What Did the Researchers Do and Find? Authors should briefly describe the study design that was used and the study’s major findings. Do include the headline numbers from the study, such as the sample size and key findings.     • What Do These Findings Mean? Authors should reflect on the new knowledge generated by the research and the implications for practice, research, policy, or public health. Authors should also consider how the interpretation of the study’s findings may be affected by the study limitations. In the final bullet point of ‘What Do These Findings Mean?’, please describe the main limitations of the study in non-technical language. The Author Summary should immediately follow the Abstract in your revised manuscript. This text is subject to editorial change and should be distinct from the scientific abstract. Please see our author guidelines for more information: https://journals.plos.org/plosmedicine/s/revising-your-manuscript#loc-author-summary* | We have now added an Author Summary following the abstract. |
| **General Editorial Request: 10** | *Introduction - please ensure that you address past research and explain the need for and potential importance of your study. Indicate whether your study is novel and how you determined that. If there has been a systematic review of the evidence related to your study (or you have conducted one), please refer to and reference that review and indicate whether it supports the need for your study.* | We have addressed past research in detail in our introduction. We have referenced our own systematic review on this subject in the introduction, which calls for further research on this subject [18]. |
| **General Editorial Request: 11** | *Discussion – please ensure that you present and organize the Discussion as follows: a short, clear summary of the article's findings; what the study adds to existing research and where and why the results may differ from previous research; strengths and limitations of the study; implications and next steps for research, clinical practice, and/or public policy; one-paragraph conclusion. Please avoid the use of sub-headings such that the discussion reads as continuous prose.* | We have reviewed our discussion and organized it as requested. We have removed the sub-headings such that the discussion reads as continuous prose. |

**REFERENCES**

1.	Liddle AD, Judge A, Pandit H, Murray DW. Adverse outcomes after total and unicompartmental knee replacement in 101,330 matched patients: a study of data from the National Joint Registry for England and Wales. Lancet. 2014;384(9952):1437-45.

2.	Hunt LP, Blom AW, Matharu GS, Kunutsor SK, Beswick AD, Wilkinson JM, et al. Patients Receiving a Primary Unicompartmental Knee Replacement Have a Higher Risk of Revision but a Lower Risk of Mortality Than Predicted Had They Received a Total Knee Replacement: Data From the National Joint Registry for England, Wales, Northern Ireland, and the Isle of Man. J Arthroplasty. 2021;36(2):471-7 e6.

3.	NJR: 18th Annual Report 2021 [Available from: https://reports.njrcentre.org.uk/Portals/0/PDFdownloads/NJR%2018th%20Annual%20Report%202021.pdf.

4.	Fitzgerald JE, Giddings CE, Khera G, Marron CD. Improving the future of surgical training and education: consensus recommendations from the Association of Surgeons in Training. Int J Surg. 2012;10(8):389-92.

5.	Sayers A, Steele F, Whitehouse MR, Price A, Ben-Shlomo Y, Blom AW. Association between surgical volume and failure of primary total hip replacement in England and Wales: findings from a prospective national joint replacement register. BMJ Open. 2020;10(9):e033045.

6.	White IR, Carlin JB. Bias and efficiency of multiple imputation compared with complete-case analysis for missing covariate values. Stat Med. 2010;29(28):2920-31.

7.	Sayers A, Whitehouse MR, Judge A, MacGregor AJ, Blom AW, Ben-Shlomo Y. Analysis of change in patient-reported outcome measures with floor and ceiling effects using the multilevel Tobit model: a simulation study and an example from a National Joint Register using body mass index and the Oxford Hip Score. BMJ Open. 2020;10(8):e033646.

8.	Fowler TJ, Aquilina AL, Reed MR, Blom AW, Sayers A, Whitehouse MR. The association between surgeon grade and risk of revision following total hip arthroplasty : an analysis of National Joint Registry data. Bone Joint J. 2022;104-B(3):341-51.

9.	Crowther MJ, Look MP, Riley RD. Multilevel mixed effects parametric survival models using adaptive Gauss-Hermite quadrature with application to recurrent events and individual participant data meta-analysis. Stat Med. 2014;33(22):3844-58.

10.	Liddle AD, Pandit H, Judge A, Murray DW. Effect of Surgical Caseload on Revision Rate Following Total and Unicompartmental Knee Replacement. J Bone Joint Surg Am. 2016;98(1):1-8.

11.	Mohammad HR, Matharu GS, Judge A, Murray DW. The Effect of Surgeon Caseload on the Relative Revision Rate of Cemented and Cementless Unicompartmental Knee Replacements: An Analysis from the National Joint Registry for England, Wales, Northern Ireland and the Isle of Man. J Bone Joint Surg Am. 2020;102(8):644-53.

12.	Jolbӓck P, Rolfson O, Cnudde P, Odin D, Malchau H, Lindahl H, et al. High annual surgeon volume reduces the risk of adverse events following primary total hip arthroplasty: a registry-based study of 12,100 cases in Western Sweden. Acta Orthop. 2019;90(2):153-8.

13.	Ravi B, Jenkinson R, Austin PC, Croxford R, Wasserstein D, Escott B, et al. Relation between surgeon volume and risk of complications after total hip arthroplasty: propensity score matched cohort study. BMJ. 2014;348:g3284.

14.	Baker P, Jameson S, Critchley R, Reed M, Gregg P, Deehan D. Center and surgeon volume influence the revision rate following unicondylar knee replacement: an analysis of 23,400 medial cemented unicondylar knee replacements. J Bone Joint Surg Am. 2013;95(8):702-9.

15.	Klasan A, Tay ML, Frampton C, Young SW. High usage of medial unicompartmental knee arthroplasty negatively influences total knee arthroplasty revision rate. Knee Surg Sports Traumatol Arthrosc. 2022;30(9):3199-207.

16.	Liddle AD, Pandit H, Judge A, Murray DW. Optimal usage of unicompartmental knee arthroplasty: a study of 41,986 cases from the National Joint Registry for England and Wales. Bone Joint J. 2015;97-B(11):1506-11.

17.	Valsamis EM, Collins GS, Pinedo-Villanueva R, Whitehouse MR, Rangan A, Sayers A, et al. Association between surgeon volume and patient outcomes after elective shoulder replacement surgery using data from the National Joint Registry and Hospital Episode Statistics for England: population based cohort study. BMJ. 2023;381:e075355.

18.	Fowler TJ, Aquilina AL, Blom AW, Sayers A, Whitehouse MR. The association between surgeon grade and implant survival following hip and knee replacement: a systematic review and meta-analysis. BMJ Open. 2021.
