# Supplementary material for: Association between surgeon training grade and the risk of revision following unicompartmental knee replacement: An analysis of National Joint Registry data
Source: PLoS Med. 2024 Sep 10;21(9):e1004445. doi: 10.1371/journal.pmed.1004445 (PMC11386457; doi:10.1371/journal.pmed.1004445)
Supplement: Attachment — Submitted filename: Response.docx [file pmed.1004445.s010.docx]

| **Reply to comments: “Association between surgeon training grade and the risk of revision following unicompartmental knee replacement: an analysis of a National Joint Registry Data” (PMEDICINE-D-24-00668R2)** | | |
| --- | --- | --- |
|  | | |
| **Editorial Comment Number** | **Comment** | **Response** |
| **Comment 1** | **GENERAL** Many of the editorial requests detailed below pertain to specific formatting and content requirements. Some may have already been incorporated into the manuscript and some may not apply, but please review the complete list of items and ensure that each item is included as necessary.  Our Academic Editor commented that your rebuttal letter was ‘thoughtful and thorough’ and only had one small suggestion at this stage: that your abstract should report absolute risks and not just relative risks. | Thank you again for taking the time to review our paper and for the detailed appraisal of our work. We were pleased to see so many positive comments from the reviewers and that all of their comments had been appropriately addressed.  We are grateful for the positive feedback on our rebuttal letter. We have addressed all comments and amended manuscript accordingly, with the exception of reporting absolute risks. We  agree that absolute risks are useful when reporting positive results. However, as this research largely shows no difference in risk across all unadjusted and adjusted analyses, we are not sure of the merit in this circumstance. In essence, there was no difference in either relative risk or absolute risk. We would prefer not to report absolute risk as we feel this could overcomplicate/introduce confusion when comprehensive results are already reported for adjusted and unadjusted analyses.    However, if the Academic Editor feels strongly that this addition to the abstract is essential, then we would prefer to report the cumulative probability of failure in each group, which is already reported in ‘All cause revision’ subsection of the methods section, Table 2, and Figure 2.  This paragraph reads as follows: *“The unadjusted cumulative probability of failure at 15 years was 17.13% (95% CI [16.44, 17.55]) for consultants, 16.42% (95% CI [14.09, 19.08]) for trainees overall…*  Alternatively we could report a restricted mean survival time, as we feel this corresponds to our original analyses and has a direct interpretation of number of days difference between the two groups. This may itself be an over complication, as it presents an additional method to report on. |
| **Comment 2** | **OBSERVATIONAL STUDIES** In the manuscript text, please indicate: (1) the specific hypotheses you intended to test, (2) the analytical methods by which you planned to test them, (3) the analyses you actually performed, and (4) when reported analyses differ from those that were planned, transparent explanations for differences that affect the reliability of the study's results. If a reported analysis was performed based on an interesting but unanticipated pattern in the data, please be clear that the analysis was data-driven. | This study is reported as per the Reporting of studies Conducted using Observational Routinely-collected health Data (RECORD) Statement. The analytical methods are clearly defined, and data driven changes in the analyses are clearly reported. Further information is given in S2 Appendix + S4 Appendix. If there are any specific recommendations, then we would be happy to address these. |
| **Comment 3** | **DATA AVAILABILITY** Please provide a URL or email address for data applications to the Census Bureau. | Please note that the authors were not required to make a data access request to the Census Bureau. All data used in this study are available from the NJR. Please see the ‘Data processing’ subsection of the Methods for clarification:  *“NJR data are annually linked to other healthcare system datasets, including Civil Registration Authority data, using unique patient identifiers. This linkage, which was carried out by the NJR prior to us obtaining the dataset, is approved by the Health Research Authority under Section 251 of the NHS act 2006 [13]. The steps taken in data processing and are summarised in the study flow diagram in Figure 1 and illustrated in greater detail in S1 Figure.”*  Please also refer to our ‘Data Availability Statement’:  *“The data used in the study are available from The National Joint Registry (NJR) (https://www.njrcentre.org.uk). Restrictions apply to the availability of these data, which were used under license for the current study, and are therefore not publicly available. Data access applications can be made to the NJR Research Committee. With NJR permission in place, the data underlying the presented results will be available to access via the NJR data access network. The authors of this manuscript are not the data owner and do not have permission to share the data.”* |
| **Comment 4** | **COMPETING INTERESTS** Thank you for acknowledging that members of the research team were funded by a contract grant from the National Joint Registry (NJR). Could you provide more details please? What does this contract entail?  The funding statement should include: specific grant numbers, initials of authors who received each award, URLs to sponsors’ websites, etc.  I see that you write that the NIHR had no role in the design and conduct of the study, etc – I would be grateful if you could include a similar statement (where it applies) with regards to the NJR. | Thanks for this comment. We have now updated the funding and competing interest statements to provide further details and clarification. This includes grant numbers where applicable.  Regarding the contract grant from the NJR: AWB, AS and MRW report holding a contract with The Healthcare Quality Improvement Partnership/The National Joint Registry in the form of the Lot 2 contract (FTS 010307-2022: Statistical Analysis, Support and Associated Services). This includes producing the NJR Annual Report.  We have now included a more general statement that reads: “The funders had no role in study design, data collection and analysis, the preparation of the manuscript, or the decision to publish.” |
| **Comment 5** | **ABSTRACT** In the final sentence of the abstract methods and findings section, please detail the limitations of the study. | We have now added the following sentence to the end of the methods and findings section.  *“Limitations of this study relate to its observational design and include: the potential for non-random allocation of cases by consultants to trainees; residual confounding; and the use of the binary variable ‘surgeon grade’, which does not capture variations in the level of experience between trainees.”* |
| **Comment 6** | **AUTHOR SUMMARY** Thank you for including this Author Summary. Line 7 – Please briefly expand on the statement that ‘there is a growing demand for surgeons to be trained in [UKR]’ – it would be useful for readers to know why there is such a demand within this summary.  Line 11 onwards – Please re-phrase your sentences here to help readers who do not have specialist knowledge to better understand exactly what you did and/or why. For example, ‘parametric survival models’ or ‘scrubbed consultants’ may not be well-understood. Please revise for accessibility to the non-scientific reader avoiding the use of what might be considered medical and/or scientific ‘jargon’.  In the final bullet point of ‘What Do These Findings Mean?’, please describe the main limitations of the study in non-technical language. Line 21 – suggest ‘These data suggest…’ or similar. | Line 7 – We have edited this point to add a more relevant and evidence-based statement. We have cited the recent BASK/EKS consensus statement. This is so relevant that we have also cited it in the first paragraph of the introduction. This point now reads as follows:  *“The British Association for Surgery of the Knee (BASK) and European Knee Society (EKS) have recommended that knee surgeons should have exposure to and training in UKR.”*  Line 11 – We have now rephrased this to read as follows:  *“We were interested in whether the procedure was performed by a fully-trained consultant surgeon, or a trainee. We were also interested in whether or not trainees were directly supervised by a consultant during the operation. The primary outcome was all cause revision surgery. We used a specialist statistical method called ‘flexible parametric survival modelling’ to analyse the data.”*  In the final bullet point, we have now added the following: *“Limitations of this study relate to its observational design. An example of a limitation is that we used a binary exposure (trainee vs. consultant), which does not capture variations in the level of experience between trainees.”* |
| **Comment 7** | **INTRODUCTION** Our journal readership is global – please consider revising the sentences here to reflect that you are referring to UK-based observations and guidelines. | Thank you for this comment. We have now edited the second sentence to read as follows:  *“The National Institute for Health and Care Excellence (NICE) recommends that patients in England and Wales with isolated medial compartment OA should be offered a choice of UKR or TKR [2].”* |
| **Comment 8** | **STATISTICAL REPORTING** A) Throughout, please quantify the main results with 95% CIs and p values. When reporting p values please report as <0.001 and where higher as p=0.002, for example. When reporting 95% CIs please separate upper and lower bounds with commas instead of hyphens as the latter can be confused with reporting of negative values.  B) Please include the actual amounts and/or absolute risk(s) of relevant outcomes (including NNT or NNH where appropriate), not just relative risks or correlation coefficients. (Example for absolute risks: PMID: 28399126). | A) We have re-reviewed the manuscript and believe that we have met the recommendations for reporting the results with 95% CIs and p-values. I can confirm that the upper and lower bounds of 95% CIs are separated with commas.  B) NB: Please note that the actual numbers of events (revision procedures), at each timepoint/for each group, are documented in detail in Table 2.  NB: Please also see our detailed response to Comment 1, for our rationale on not including absolute risks. If the Academic Editor, feels that this is an essential addition then we would welcome further feedback. |
| **Comment 9** | **DISCUSSION** Please re-phrase ‘We included over 100,000 knees…’ to ‘We included over 100,000 knee operations…’ (or similar). | We agree that needs editing. We have re-phrased this sentence to read as follows:  *“We included over 100,000 UKRs…”* |
| **Comment 10** | **TABLES and FIGURES** Throughout, including the supporting files, please provide titles/captions/footnotes which clearly describe the table/figure content without the need to refer to the text. Please ensure all abbreviations including those used for statistical reporting are also clearly defined in the footnote. Throughout please indicate whether your analyses are adjusted or unadjusted and where adjusted analyses are presented please also present unadjusted analyses for comparison. Please also ensure to clearly detail in the footnote/caption the factors which are adjusted for. Please refer to <https://journals.plos.org/plosmedicine/s/figures#loc-pages> for further guidance. Please consider avoiding the use of green and/or red to make your figures more accessible to those with colour blindness. | We have reviewed all tables and figures to ensure that these guidelines have been met. |
| **Comment 11** | **REFERENCES** In the bibliography please ensure that you list up to but no more than 6 author names followed by et al. For all web references please ensure you include an, ‘Accessed [date].’ Journal name abbreviations should be those listed in the National Center for Biotechnology Information (NCBI) databases. Please review your reference list to ensure that it is complete and correct. If you have cited papers that have been retracted, please include the rationale for doing so in the manuscript text, or remove these references and replace them with relevant current references. Any changes to the reference list should be mentioned in the rebuttal letter that accompanies your revised manuscript. For further guidance, please see <https://journals.plos.org/plosmedicine/s/submission-guidelines#loc-references> | We have re-reviewed the references section and ensured that the references all meet these recommended guidelines. |
| **Comment 12** | **MAIN TEXT - MISCELLANEOUS** Throughout your manuscript, please avoid the use of the term ‘retrospective’ to describe your study and instead refer to it as ‘observational’. In the Abstract, for example. Please check and amend throughout.  Line 245, 261, 271 – Please remove the data availability, funding and conflict of interest statement from the main manuscript and include these only in the manuscript submission form when you re-submit.  Line 280 – please move the ethics statement to the methods section of the main manuscript. | We have now edited the manuscript to replace the term ‘retrospective’ with ‘observational’ throughout.  The data availability, funding, and conflict of interest statements have now been removed from the main manuscript and included in the submission form on resubmission.  The ethics statement has been moved to the methods section of the main manuscript as per your request |
| **Comment 13** | **SUPPORTING INFORMATION** Please ensure you apply all guidance detailed above to the supporting information files. Please cite your Supporting Information as outlined here: <https://journals.plos.org/plosmedicine/s/supporting-information> In the published article, supporting information files are accessed only through a hyperlink attached to the captions. For this reason, you must list captions at the end of your manuscript file. You may include a caption within the supporting information file itself, as long as that caption is also provided in the manuscript file. Do not submit a separate caption file. Please ensure that all guidance above is applied as relevant to the supporting files. | The supplementary methods and figures have now been described as follows: S1 Appendix, S1 Figure, etc. We have edited titles and references in the text accordingly and made it clear where changes have been made.  The caption list at the end of the manuscript has been amended accordingly. |
| **Comment 14** | **SOCIAL MEDIA** To help us extend the reach of your research, if not already done so, please detail any X (formerly Twitter) handles you wish to be included when we tweet this paper (including your own, your coauthors’, your institution, funder, or lab) in the manuscript submission form when you re-submit the manuscript. | While none of the authors are active on X, the National Joint Registry handle is @JointRegistry. It may also be appropriate to include @Brms_Research, which is the handle for Bristol Medical School Research. |
