# Supplementary material for: Association between surgeon training grade and the risk of revision following unicompartmental knee replacement: An analysis of National Joint Registry data
Source: PLoS Med. 2024 Sep 10;21(9):e1004445. doi: 10.1371/journal.pmed.1004445 (PMC11386457; doi:10.1371/journal.pmed.1004445)
Supplement: Attachment — Submitted filename: Response.docx [file pmed.1004445.s011.docx]

| **Reply to comments: “Association between surgeon training grade and the risk of revision following unicompartmental knee replacement: an analysis of National Joint Registry Data” (PMEDICINE-D-24-00668R3)** | | |
| --- | --- | --- |
|  | | |
| **Editorial Comment Number** | **Comment** | **Response** |
| **Comment 1** | I have discussed your response to ‘Comment 1’ with the academic editor and he would be happy for you to report the cumulative probabilities in your abstract. | We are grateful that you have considered this and we have now quoted the unadjusted cumulative probability of failure at 15 years, as follows:  “The unadjusted cumulative probability of failure at 15 years was 17.13% (95% CI [16.44, 17.85]) for consultants, 16.42% (95% CI [14.09, 19.08]) for trainees overall, 15.98% (95% CI [13.36, 19.07]) for trainees supervised by a scrubbed consultant, and 17.32% (95% CI [13.24, 22.50]) for trainees not supervised by a scrubbed consultant.” |
| **Comment 2** | Title: Please revise the latter part of your title for clarity, e.g. replace ‘an analysis of a National Joint Registry Data’ with ‘an analysis of National Joint Registry Data’ or similar. | Thank you for this. This was an unfortunate typo. We have amended this. |
| **Comment 3** | Please mention the location (i.e. England and Wales) in the Background section of the Abstract. | We have edited the Background section of the Abstract to read as follows:  “Unicompartmental knee replacements (UKRs) are performed by surgeons at various stages in training with varying levels of supervision, but we do not know if this is a safe practice with comparable outcomes to consultant-performed UKR. The aim of this study was to use registry data for England and Wales to investigate the association between surgeon grade (consultant, or trainee), the senior supervision of trainees (supervised by a scrubbed consultant, or not), and the risk of revision surgery following UKR.”  We are happy for this to read ‘for England and Wales’, or ‘from England and Wales’ according to editorial preference. |
| **Comment 4** | We suggest that you revise the statement in your abstract that reads ‘Unsupervised trainee cases were associated with an increased risk of revision for unexplained pain compared to consultant-performed UKRs, in all but the fully adjusted model’. Given that there was no difference in the fully adjusted model, it would seem to us that essentially there was no real difference to be found here. | We have deleted this sentence from the abstract. We agree that this is not a main finding, with essentially no difference. It is addressed appropriately in the Results/Discussion sections. |
| **Comment 5** | Please replace ‘all cause’ with ‘all-cause’ (i.e. with a hyphen) throughout your manuscript. | We have replaced this throughout the manuscript, including tables and legends. |
| **Comment 6** | Thank you for revising the phrase ‘scrubbed consultant’ in line 11 of your earlier version of the manuscript. Please revise the phrase in the Methods and Findings section of your abstract too. | We have now edited the Methods and findings section of the abstract to read as follows:  “Exposures were the grade of the operating surgeon (consultant, or trainee) and whether or not trainees were directly supervised by a consultant during the procedure (referred to as ‘supervised by a scrubbed consultant’).” |
| **Comment 7** | In the conclusions section of your abstract, please insert an apostrophe after the word ‘years’ in the phrase ‘16 years follow up’, so it reads ‘16 years’ follow up’. | This has been amended. For consistency, we have made the same amendment in line 1 of the Discussion. And also in the Conclusions subsection of the Discussion. |
| **Comment 8** | Under your Exposures subheading, you mention ‘scrubbed consultants’. This is completely acceptable here, but it might make better reading if you qualify what this term means in the first instance of mentioning it and then state something along the lines of ‘henceforth referred to as scrubbed consultants…’ (or similar). | Thank you for this comment. We have now amended this paragraph to read as follows:  “The secondary exposure was whether or not trainees were directly supervised by a consultant during the procedure (exposure B). We refer to direct consultant supervision as ‘supervised by a scrubbed consultant’ throughout this paper. Therefore, trainee cases were subcategorised as follows: 1) trainee supervised by a scrubbed consultant, or 2) trainee not supervised by a scrubbed consultant. Cases were categorised as ‘supervised by a scrubbed consultant’ if the first assistant was recorded as a consultant.” |
| **Comment 9** | On page 11 of your manuscript, replace the square brackets for ASA, IMD and BMI with parentheses, i.e. (). | We have edited this accordingly. |
| **Comment 10** | On page 12, line 6, there is a missing word ‘in’. The sentence ‘…more were they involved the design’ should have an ‘in’ before the word ‘the’. | We have edited this accordingly. |
| **Comment 11** | In the section named Contributors, I note that the initials used for the authors used is not consistent with those used in the Financial Disclosures section – could this be revised for consistency please? | We have revised these initials, which are now consistent throughout. |
| **Comment 12** | In your Author Summary, could you briefly expand here why UKR is better than the more usual alternative; why is this recommended by NICE, etc? | We have now added the following as the second bullet point in the Author Summary,  “Proposed advantages of UKR over TKR include superior functional outcomes, reduced length of stay, fewer medical complications, greater cost-effectiveness, and lower mortality. However, UKR revision rates are considerably higher than primary TKR revision rates.”  Please note that this same statement is made in the introduction, where it is included with appropriate citations. |
